# Analysis of metabolites and metabolism-mediated biological activity assessment of ginsenosides on microfluidic co-culture system

**DOI:** 10.3389/fphar.2023.1046722

**Published:** 2023-01-30

**Authors:** Zhongyu Li, Jiwen Li, Mei Sun, Lei Men, Enhua Wang, Yiran Zhao, Keke Li, Xiaojie Gong

**Affiliations:** ^1^ College of Life Science, Dalian Minzu University, Dalian, China; ^2^ School of Biological Engineering, Dalian Polytechnic University, Dalian, China

**Keywords:** ginsenosides, drug metabolism, biological activity, microfluidic, organ-on-chip, drug evaluation

## Abstract

*In vivo*, the complex process of drugs metabolism alters the change in drug composition and determines the final pharmacological properties of oral drugs. Ginsenosides are primary constituents of ginseng, whose pharmacological activities are greatly affected by liver metabolism. However, the predictive power of existing *in vitro* models is poor due to their inability to mimic the complexity of drug metabolism *in vivo*. The advance of organs-on-chip-based microfluidics system could provide a new *in vitro* drug screening platform by recapitulating the metabolic process and pharmacological activity of natural product. In this study, an improved microfluidic device was employed to establish an *in vitro* co-culture model by culturing multiple cell types in compartmentalized microchambers. Different cell lines were seeded on the device to examine the metabolites of ginsenosides from the hepatocytes in top layer and its resulting efficacy on the tumors in bottom layer. Metabolism dependent drug efficacy of Capecitabine in this system demonstrated the model is validated and controllable. High concentrations of CK, Rh2 (S), and Rg3 (S) ginsenosides showed significant inhibitory effects on two types of tumor cells. In addition, apoptosis detection showed that Rg3 (S) through liver metabolism promoted early apoptosis of tumor cells and displayed better anticancer activity than prodrug. The detected ginsenoside metabolites indicated that some protopanaxadiol saponins were converted into other anticancer aglycones in varying degrees due to orderly de-sugar and oxidation. Ginsenosides exhibited different efficacy on target cells by impacting their viabilities, indicating hepatic metabolism plays an important role in determining ginsenosides efficacy. In conclusion, this microfluidic co-culture system is simple, scalable, and possibly widely applicable in evaluating anticancer activity and metabolism of drug during the early developmental phases of natural product.

## Introduction

Malignant tumors are severe threats to human health and a major cause of death worldwide (Zhang et al., 2021). In 2020, 19.3 million malignant tumor cases and nearly 10 million malignant tumor deaths were reported worldwide, with 11.7% of female breast cancer, 11.4% of lung cancer, and 10.0% of colorectal cancer being the most common (Sliwinski et al., 2022). In spite of numerous clinical studies, the benefits of chemotherapeutic agents for the treatment of cancer are unclear. Natural product is a good source for discovering natural drugs and drug precursor structures with unique advantages in antitumor therapy (Efferth et al., 2008). *In vivo*, the complex process of drugs metabolism alters the change in drug composition and determines the final pharmacological properties of oral drugs. The liver is an important organ for drug metabolism, with a crucial influence on the final pharmacological action of drugs. Many drugs exhibit altered biological activity after liver metabolism. For example, capecitabine is the first-line drug for breast cancer treatment in clinical settings, but it exhibits low pharmacological activity *in vitro*. Effective chemotherapy for tumors requires pharmacological activity after liver metabolism.

Although hepatic metabolism plays a crucial role in determining drug biological activity, it is difficult to consider it in early-phase drug development due to sparse recapitulation of the functional responses of hepatic metabolism in existing drug evaluation models. The animal model is commonly used to study drug metabolism. However, due to varied species and metabolic features, the results are often different from that of human beings (Barré-Sinoussi and Montagutelli, 2015). The traditional two-dimensional culture *in vitro* can moderately replace the animal model, but it cannot replicate the physiological environment of cells *in vivo* (Cong et al., 2020). An *in vitro* model that can evaluate biological activity with a physiologically relevant process is greatly needed. Organs-on-chip-based microfluidics system provides a new technological platform for similar studies. Recently, a variety of liver models have been established for such studies. For example, the liver chip can evaluate liver metabolisms and aid in determining the first pass effect by integrating an intestine-like structure on microfluidic system (Choe et al., 2017). The anticancer activity of luteolin was evaluated using the microfluidic system combined with liver and tumor as a PK–PD model (Lee et al., 2017). The results showed that the anticancer activity on chip was significantly weaker than that in two-dimensional culture. In previous study, a new multilayer microfluidic chip was developed by our group that effectively evaluated the metabolism, toxicity, and efficacy of drugs in different cells simultaneously (Li et al., 2016). This novel chip system enables the assessment of complex metabolic processes and anticancer activity of drug on a single device. In recent years, organ-on-chip technology has attracted an increasing interest for drug screening and evaluation. The U.S. House of Representatives passed the Food and Drug Amendments of 2022 (H.R. 7,667–Food and Drug Amendments of 2022), officially incorporating organ chips into the non-clinical drug trials.

Ginseng has a good medicinal value as a valuable traditional medicine, with unique advantages in antitumor therapy (Chen et al., 2020). Ginsenosides are the main active components of Panax ginseng, possessing rich pharmacological effects. However, only few studies have reported their effect of liver metabolism on anticancer activity. In recent studies, ginsenosides have shown that they may form new metabolites after metabolism, changing their biological activity (Cao et al., 2020). For example, in rat gastrointestinal tract, Rg3 ginsenoside degrades successively into Rh2 and protopanaxadiol aglycone (PPD). It has higher plasma exposure levels and stronger antitumor activity than that of Rg3 alone (Hao et al., 2010). Although significant advances have been made, systematic studies for hepatic metabolism and biological activity of ginsenosides *in vitro*, which may provide the theoretical basis for rational anticancer application and scientific development of ginsenosides, has not been attempted yet.

In the present study, a liver–tumor co-culture model was constructed based on the previous multilayer microfluidic system. The developed microfluidic chip consists of compartmentalized microfluidic microchambers to culture different types of cells. In this chip added viewing window, hepatic cells were cultured in the top chamber to simulate liver tissues for drug metabolism, and three types of cells were cultured in the compartmentalized bottom channels representing different tumor and normal tissues. Re, CK, Rh2 (S), and Rg3 (S) ginsenosides metabolized from hepatic cells were monitored and analyzed, and the effects of these ginsenosides were assessed on various cells. In the integrated chip, liver changed the biological activity of some drugs on different cells, which was a significant effect. We explored the effects of key factors, such as liver metabolism, on the biological activity of ginsenosides. This study demonstrated the utility of the compartmentalized microdevice in natural product for anticancer activity testing, facilitating the drug discovery and drug screening applications in a biomimetic model in a simple and reliable manner.

## Materials and methods

### Materials

Polycarbonate porous membrane (Whatman, UK), Polydimethylsiloxane (PDMS, Dow Corning, USA), SU-8 3035 negative photoresist (MicroChem, USA), Dulbecco’s Modified Eagle Medium (DMEM, Gibco, USA), fetal bovine serum (FBS, Gibco, USA), trypsin and EDTA (Gibco, USA), rat tail type-I collagen (BD, USA), Calcein/PI cell viability assay kit (Beyotime, China), cell counting kit-8 (CCK-8, APExBIO, USA), Annexin V-FITC apoptosis detection kit (Beyotime, China), Capecitabine (CAP, purity≥98%, Sigma, USA), Re, Rg3(S), Rh2(S) and CK ginsenosides (purity≥98%) were purchased from Sichuan China. All chemical reagents were mass spectrometry reagent grade.

### Design and fabrication of microfluidic device

**FIGURE 1:**
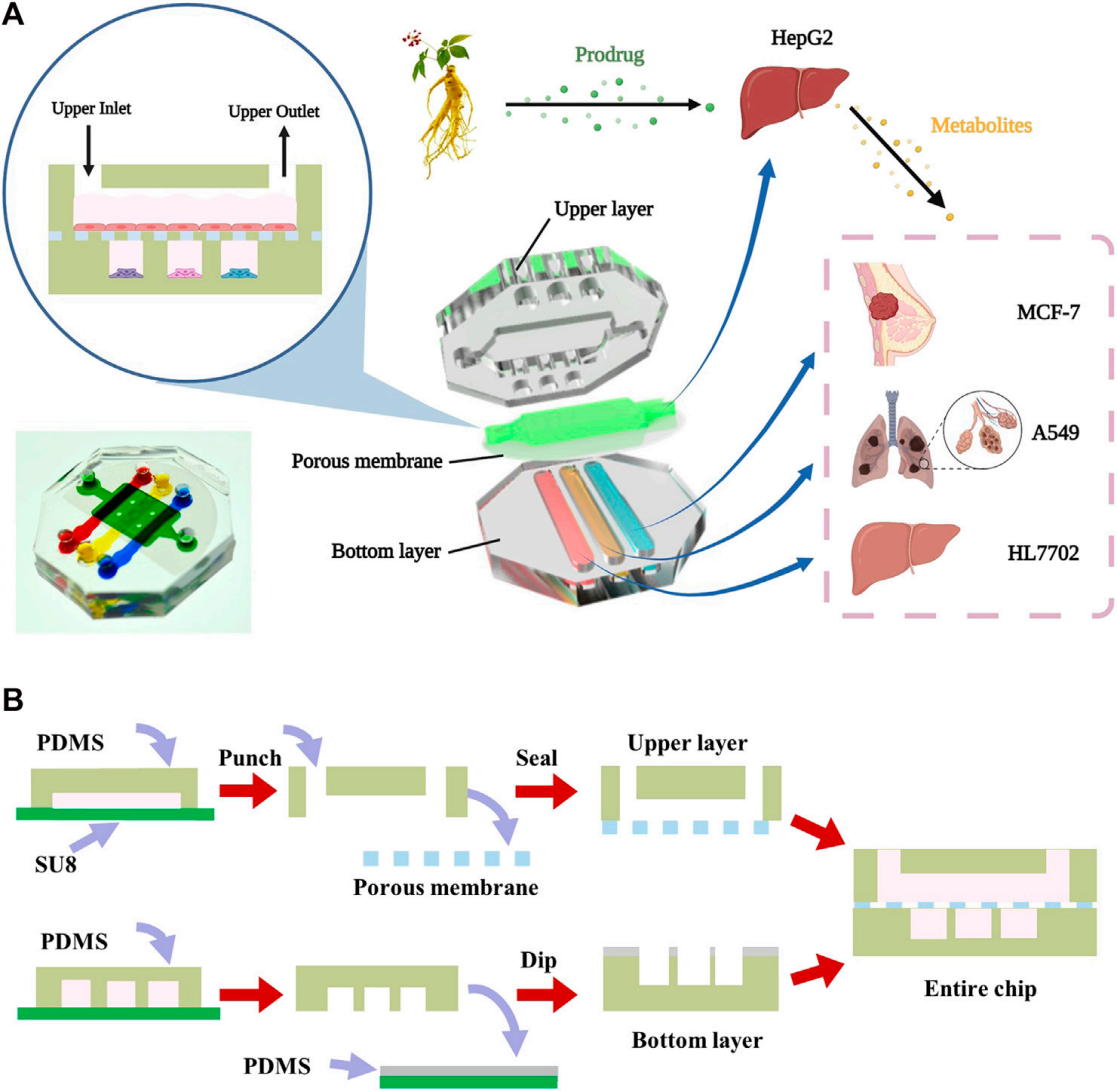
Design and assembly of the multilayer chip **(A)** The schematic diagram of the structure of multilayer microfluidic chip device including the upper layer, middle porous membrane and bottom layer. **(B) **The fabrication process of the multilayer microdevice.

The microfluidic device was developed to establish a two-layer organs-on-chip on the basis of conventional microfabrication technique (Figure 1B). The microfluidic chip consists of two layers of PDMS separated by a porous membrane with 0.4 μm pore size, which allowing the transition of small molecular compounds. The upper layer of the chip had one cell culture chamber which was 10 mm in length, 6 mm in width and 500 μm in height, and the bottom layer had three microchannels, each of which was 15 mm in length, 2 mm in width and 500 μm in height. Each chamber was connected to inlet and outlet for in and out sampling, respectively.

The multilayer chip was fabricated using soft lithography and micromolding technology as previously reported (Wen et al., 2014; Wen et al., 2015; Zhu et al., 2016). SU-8 photoresist was spin-coated onto two clean silicon wafers and selectively cured exposed to ultraviolet light source with different masks to prepare the template. The mold was developed in ethyl lactate, followed by hard bake in 180°C for 2 h. Next, a mixture of the uncured polydimethylsiloxane monomer and curing reagent at a ratio of 6:1 (v/v) poured onto the mold to generate two PDMS layer replicas. Finally, the PDMS replicas were sealed together with the middle porous membrane (B. Chueh et al., 2007; Aran et al., 2010).

### Co-culture of cell on chip

Four types of human cells were used: HepG2, A549, MCF-7, and HL7702 cells. All the cells were cultured in DMEM supplemented with 10% FBS, and 1% (v/v) penicillin-streptomycin in a cell incubator at 37°C, 5% CO_2_. The cells were passaged weekly at confluence with change in medium every 48 h. The maximal times of cell passage were less than 10 (<3 months) after the cells were recovered from frozen stocks.

Following fabrication, the chips were exposed to UV overnight to get sterilized. The chambers of the chip were treated with type-I collagen (8 μg/mL) for 12 h to maintain a biocompatible surface. Four types of cells were trypsinized and detached, followed by centrifugation and re-suspension of the cells in the medium to obtain a required density of 1×10^6^ cells/mL. HepG2 cells were perfused into the upper chamber and cultured. After adherent growth of HepG2 cells, the suspended A549, MCF-7, and HL7702 cells were injected into the bottom chambers from the respective inlets. The chip was then incubated in the incubator in a humid atmosphere at 37°C, 5% CO_2_ for at least 6 h to enable cell adherence.

### Cell viability and apoptosis assay

The experiment was divided into HepG2 (–) and HepG2 (+) groups. DMEM media containing Re, CK, Rh2 (S), and Rg3 (S) ginsenosides were added into the upper culture chambers. Ginsenosides were converted into metabolites by the hepatic cells in the top layer and then diffused into the lower cell chambers where the target cells were stimulated by the drugs and their metabolites. The concentrations of ginsenosides are shown in Table 1. After 24 h of ginsenoside stimulation, the cell toxicity in the bottom chambers was analyzed using Calcein/PI cell viability assay kit. Dead cells were stained red, whereas the viable cells were stained green. Quantitative data on cell vitality were tested using the CCK-8 assay. A 50 μL cell culture medium with 10% CCK-8 reagent was added to the bottom chambers to analyze A549, MCF-7, and HL7702 cells. The solutions were collected into a 96-well plate, and their absorbances were measured using a microplate reader at 450 nm.

**TABLE 1:** The concentration of ginsenosides for cell viability assay.

Name of the drug	Low ( μ M)	Drug concentration	High ( μ M)
		Medium ( μ M)	
CAP	50	100	200
CK	25	50	100
Re	50	100	200
Rh2(s)	25	50	100
Rgs3(s)	25	50	100

Cell apoptosis was assayed using the Annexin V-FITC apoptosis detection kit. Briefly, HepG2 (–) and HepG2 (+) groups were treated with DMEM media containing 0.5% DMSO and ginsenosides, respectively. The concentrations of ginsenosides are shown in Table 2. In total, 5 × 10^5^ cells were collected and washed twice using PBS. Cells were centrifuged at 1,000 g for 5 min at 4°C. The cells were then resuspended in 195 μL of binding buffer and incubated with 1 μL of Annexin V-FITC and 5 μL of PI staining solution for 15–20 min at room temperature. Cell apoptosis was measured using flow cytometry.

**TABLE 2:** The concentration of ginsenosides for determination of apoptosis.

Name of the drug	A549	Cell line	HL7702
		MCF-7	
CK	50	50	50
Re	200	200	200
Rh2(s)	100	50	50
Rgs3(S)	100	100	100

### Ginsenoside metabolite analysis using UPLC-MS

HepG2 (–) and HepG2 (+) groups were treated with CK (50 μM), Rh2 (S) (50 μM), and Rg3 (S) (100 μM), respectively. The culture medium in the bottom chambers was collected after 24 h for detection of ginsenoside metabolites using UPLC-MS. A total of 1 mL culture medium containing metabolite was added to 3 mL methanol and vortexed for 2 min. After centrifuging at 10000 g for 10 min, 2.5 mL of the upper layer was transferred into a polythene tube and dried at 37°C under a gentle stream of nitrogen. The dried residue was re-dissolved in 600 μL methanol solution. The methanol solution was filtered through 0.22 μM filter membranes. Finally, a 5 μL aliquot was injected into the UPLC-MS for analysis.

The UPLC-MS analysis was performed using a Waters ACQUITY™ ultra-performance liquid chromatography system coupled with an LTQ-Orbitrap Elite mass spectrometer equipped with an ESI ion source in negative ion mode. Chromatographic column: ACQUITY UPLC™ HSS T3 (2.1 mm × 100 mm, 1.8 μm). Mobile phase: A phase is an aqueous solution containing 0.1% formic acid, B phase is acetonitrile; gradient elution of A and B, 0–2 min, 5%–12% B; 3.2 min, 30% B; 9 min, 40% B; 12 min, 70% B; 13 min, 100% B; 18 min, 100% B; 23 min, 5% B; and 25 min, 5% B. Flow rate: 0.2 mL min^−1^; column temperature: 60°C; sample room temperature: 20°C; sample volume: 5 μL. UV 203 nm was on. The mass spectrometry operated in negative ion data. The scan range for full MS was set from m/z 100–1,200 using FT mass analyzer with a 120000 resolution.

### Image and statistical analysis

 Illustrations in this manuscript were created and assembled with BioRender (https://biorender.com) and Adobe Illustrator 2021. Statistical analysis was performed using one-way ANOVA and multiple t-test. All of experiments were performed at least in triplicates and all of the data were presented as means ± standard error. The expression method of statistical difference was as follows: *p < 0.05, **p < 0.01, ***p < 0.001.

## Results

### Design of multilayer and multifunctional chip

A two-layer microdevice was designed and fabricated to characterize the transhepatic metabolic process of ginsenosides *in vitro*, followed by an assessment of its drug activity on target cells. HepG2 cells representing the liver were seeded on the upper layer of the chamber. A549 (lung cancer), MCF-7 (breast cancer), and HL7702 (liver normal cells) cell lines were seeded into separate microchambers in the bottom layer to investigate the drug efficacy of ginsenosides on different type of cells (Figure 1A). The multilayer design of the chip facilitated the culture of different cell types in compartmentalized microchambers and characterized drug metabolism-dependent bioactivity on various target cells in a single assay. The device consisted of a upper layer for culture of liver cells and a bottom layer with compartmentalized microchambers for culture of cancer and normal tissue cells. A porous membrane was sandwiched between the two layers to transport drug metabolites from the liver cells in the top layer to different target cells in the bottom layer. The chip added viewing window on demand to facilitate the downstream analysis of organ-specific cells.

### Effects of ginsenosides on viability of three cells

Capecitabine (CAP) is a prodrug for the treatment of cancer. It has no pharmacological activity and can be converted into 5-fluorouracil (5-FU) by liver metabolism, which shows cytotoxicity (Sakai et al., 2022). Using CAP as the positive control of cell viability experiment, the results showed that the inhibitory effect of CAP on three kinds of cells was dependent on liver metabolism, which was consistent with the reports of *in vivo* studies, and proved the effectiveness and controllability of this model in metabolic research.

**FIGURE 2:**
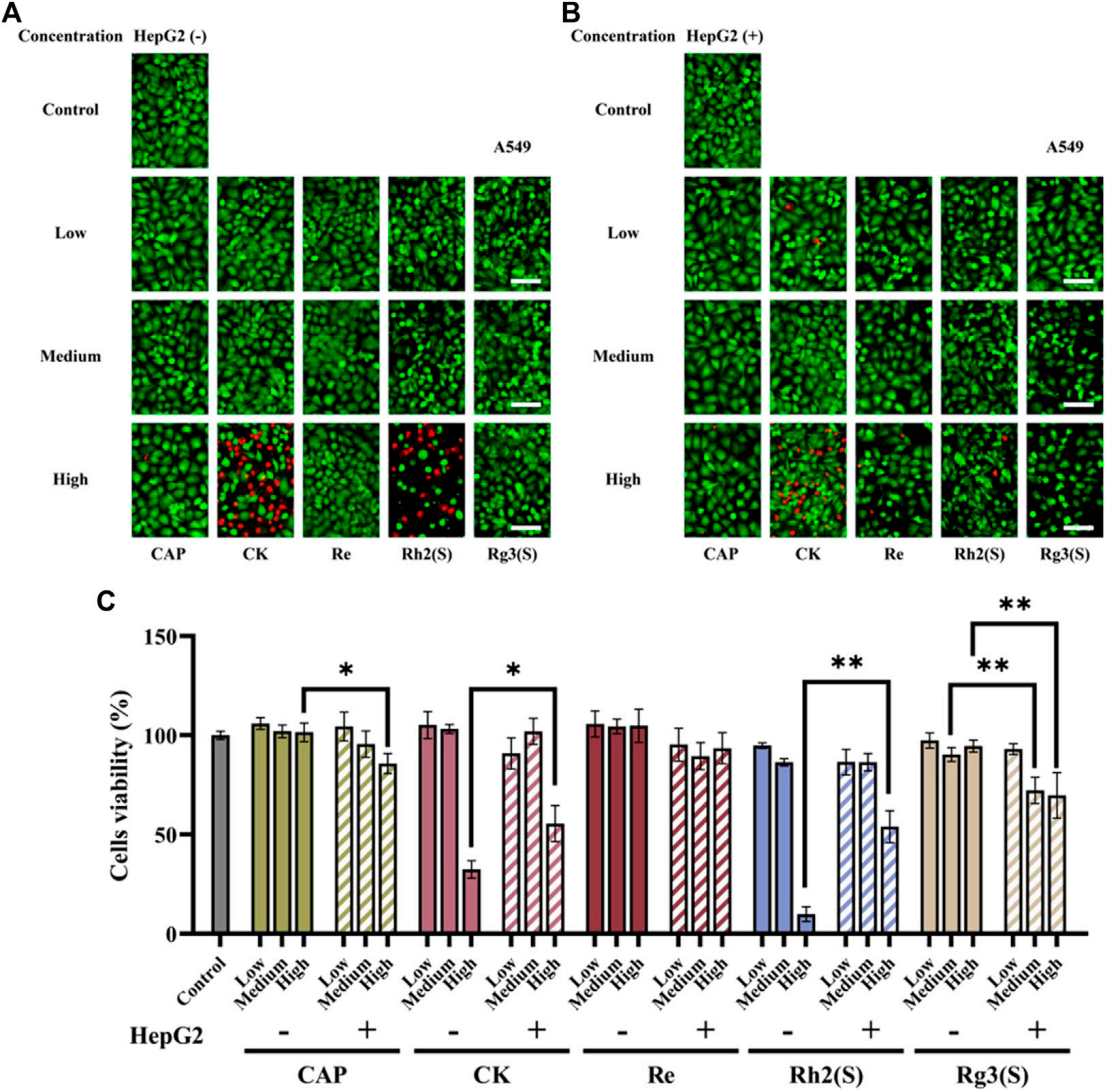
Effects of different ginsenosides on the viability of A549 cells. Fluorescent image of different concentrations of ginsenosides acting on A549 cells without **(A)** and with **(B) **HepG2 cells. Scale bar, 100 μm. **(C)** Quantitative assay of cell viability of A549 cells treated with different concentrations of ginsenosides. All of experiments were performed at least in triplicates and all of the data were presented as means ± standard error.

Figure 2C shows the effects of high, medium, and low concentrations of ginsenosides on the viability of A549 cells after 24 h. Using CAP as a positive control, fluorescence and cell viability quantitative maps showed that Re had no significant inhibitory activity on A549 cells regardless of their metabolization through the liver. High concentrations of CK and Rh2 (S) showed strong antitumor activity before liver metabolism, and the cell survival rates of A549 cells were 32.4% ± 4.36% and 9.8% ± 3.67%, respectively. After liver metabolism, the antitumor activity of the two ginsenosides decreased, and the survival rate of A549 cells increased to 55.5% ± 9.06% and 53.9% ± 8.01%, respectively. The corresponding fluorescence map directly reflected this result (Figures 2A,B). In contrast, Rg3 (S) had no significant inhibitory effect on A549 without liver metabolism, and the cell survival rate was 94.5% ± 2.98% after A549 cells were treated with high concentrations of Rg3 (S). However, after liver metabolism, the antitumor activity of medium and high concentrations of Rg3 (S) was significantly enhanced, and the cell survival rate was reduced to 72.1% ± 6.65% and 69.7% ± 11.48%. Although the antitumor activity of CK and Rh2 (S) was opposite to that of Rg3 (S) after liver metabolism, the antitumor activity of the former was still stronger than that of the latter.

**FIGURE 3:**
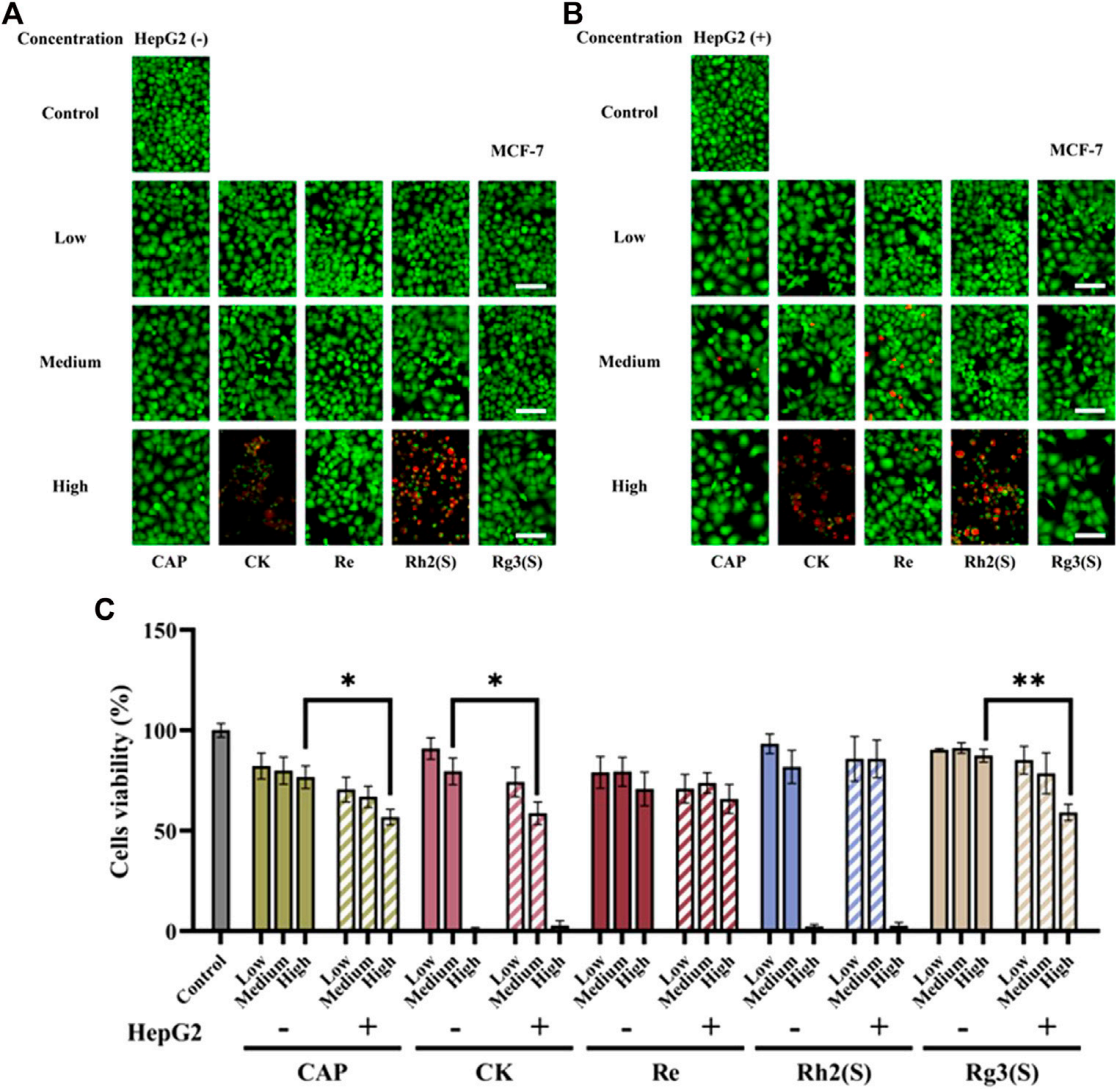
Effects of different ginsenosides on the viability of MCF-7 cells. Fluorescent image of different concentrations of ginsenosides acting on MCF-7 cells without **(A)** and with **(B)** HepG2 cells. Scale bar, 100 μm. **(C)** Quantitative assay of cell viability of MCF-7 cells treated with different concentrations of ginsenosides. All of experiments were performed at least in triplicates and all of the data were presented as means ± standard error.

As shown in Figures 3A–C, the results of MCF-7 cells were similar to those of A549 cells. Re had no significant inhibitory effect on MCF-7 cells regardless of their metabolization through the liver. High concentrations of CK, Rh2 (S), and Rg3 (S) showed strong antitumor activity, and their inhibitory effect on MCF-7 cells was stronger than that on A549 cells. After medium concentration of CK was metabolized by the liver, the cell survival rate decreased from 79.6% ± 6.62%–58.7% ± 5.62%, and the antitumor activity was enhanced. Similar to A549 cells, Rg3 (S) showed low toxicity to MCF-7 cells, the inhibitory effect significantly improved after liver metabolism, and the cell survival rate of MCF-7 cells decreased from 87.4% ± 3.16%–59.1% ± 4.09%.

**FIGURE 4:**
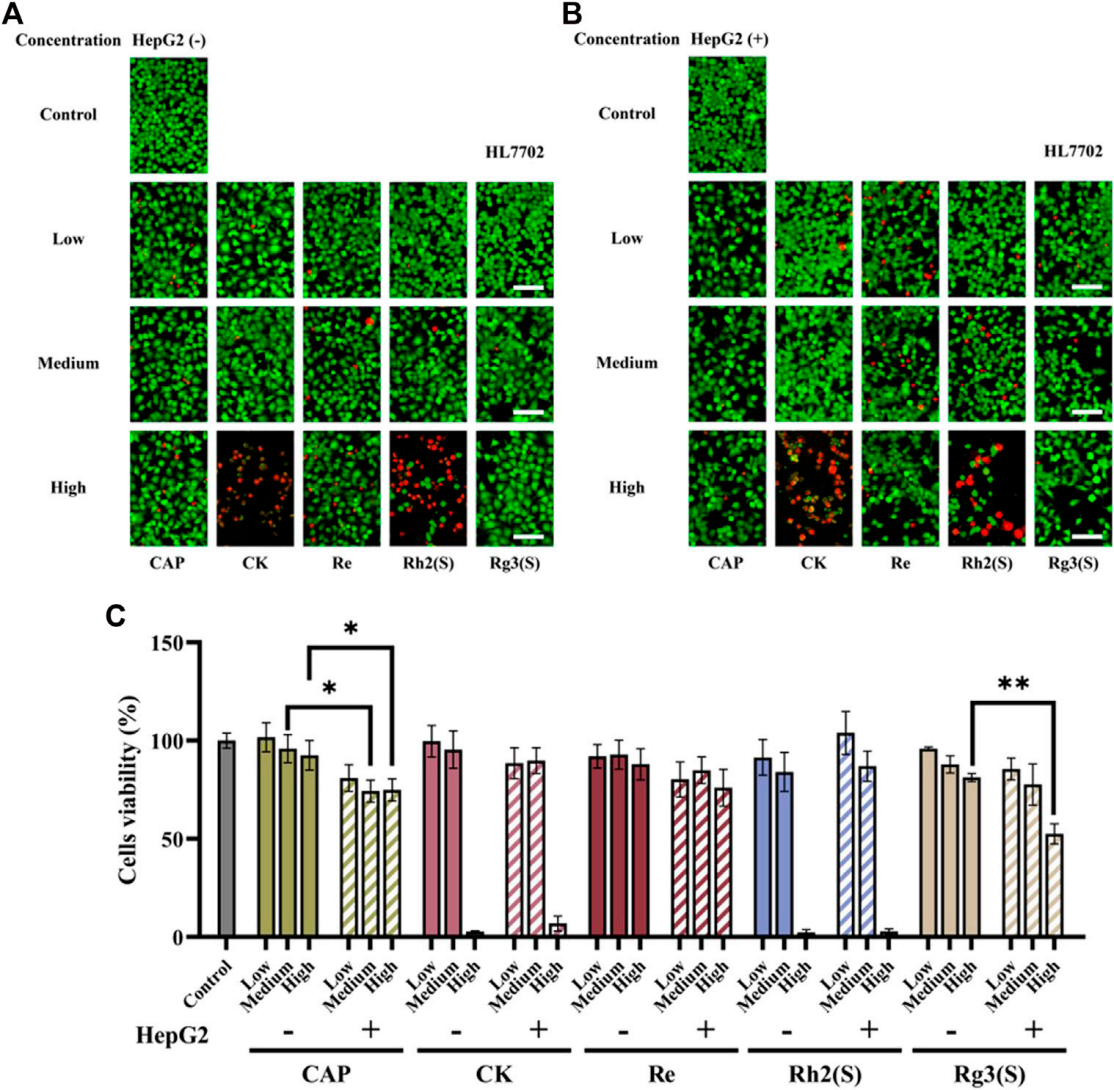
Effects of different ginsenosides on the viability of HL7702 cells. Fluorescent image of different concentrations of ginsenosides acting on HL7702 cells without **(A)** and with **(B)** HepG2 cells. Scale bar, 100 μm. **(C)** Quantitative assay of cell viability of HL7702 cells treated with different concentrations of ginsenosides. All of experiments were performed at least in triplicates and all of the data were presented as means ± standard error.

The effect of different ginsenosides on HL7702 was consistent with that of MCF-7 cells (Figures 4A–C), except that Rg3 (S) showed a specific inhibitory activity on HL7702 before and after liver metabolism in a dose-dependent manner. The inhibitory effect of CK on HL7702 did not improve significantly after liver metabolism.

### Effects of ginsenosides on apoptosis of different target cells

**FIGURE 5:**
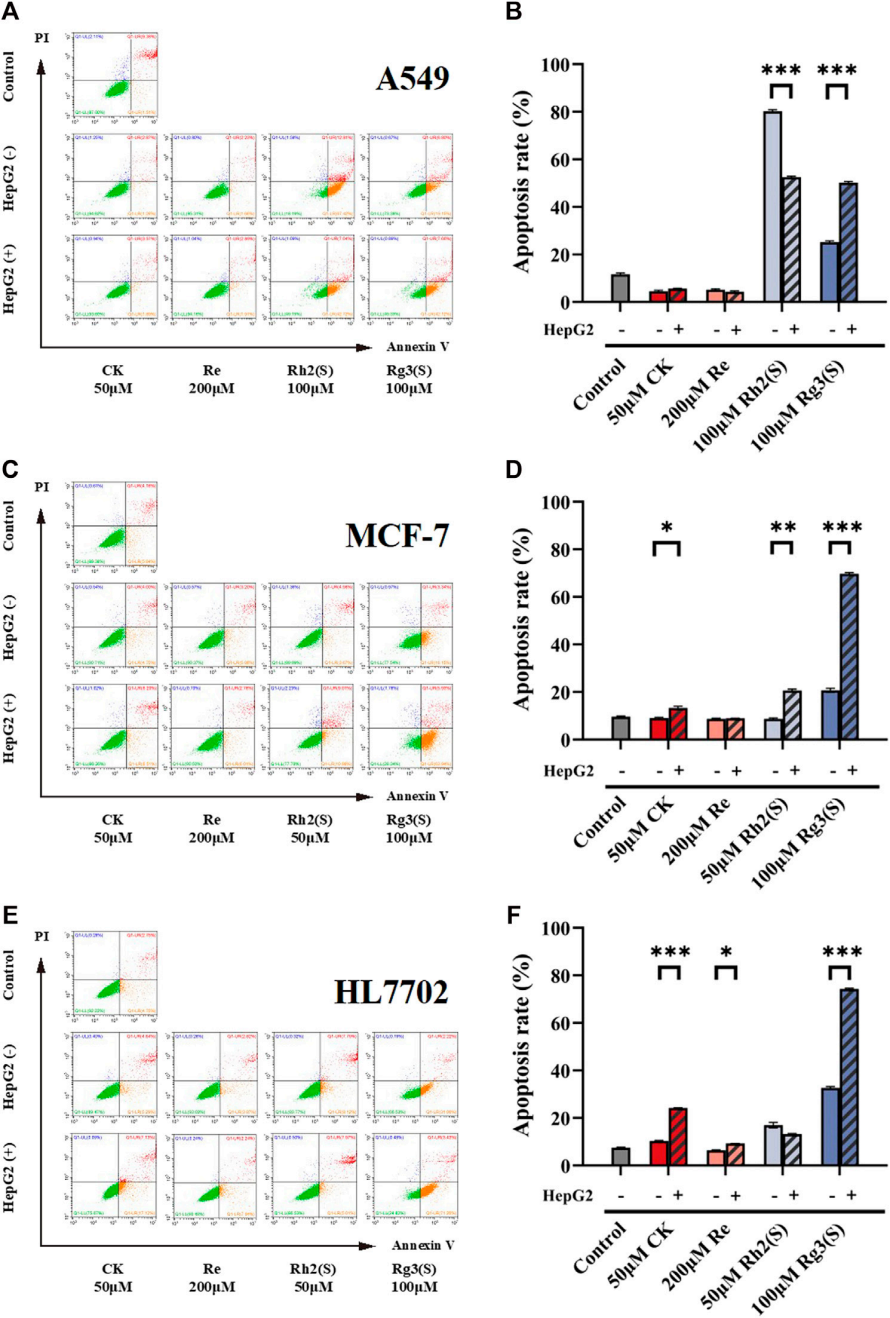
Effect of ginsenosides on apoptosis of different target cells. Apoptosis of A549 cells was measured by flow cytometry **(A,B)** quantitative assay of apoptosis. Apoptosis of MCF-7 cells was measured by flow cytometry **(C,D)** quantitative assay of apoptosis. Apoptosis of HL7702 cells was measured by flow cytometry **(E,F)** quantitative assay of apoptosis. All of experiments were performed at least in triplicates and all of the data were presented as means ± standard error.

Different ginsenosides acted on target cells without or through liver metabolism, and apoptosis was detected. The cell viability quantitative map of Figure 2C shows that a significant difference was observed in the antitumor activity of high concentration Rh2 (S) before and after liver metabolism. The survival rate of A549 cells before and after liver metabolism was 9.8% ± 3.67% and 53.8% ± 8.01%, respectively. To explore whether apoptosis is related to the difference, the amount of A549 cell apoptosis treated with a high concentration of Rh2 (S) for 24 h was detected. A high concentration of Rh2 (S) without liver metabolism in A549 cells reported an early and late apoptosis rates of 67.42% ± 0.60% and 12.81% ± 0.66%, respectively. Similarly, a high concentration of Rh2 (S) through liver metabolism in A549 cells reported a reduced early and late apoptosis rates of 42.72% ± 0.34% and 7.04% ± 0.37%, respectively (Figure 5A). After hepatic metabolism, a high concentration of Rh2 (S) inhibited the early apoptosis of A549 cells and reduced the apoptosis rate. Rg3 (S) without liver metabolism in A549 cells reported an early and late apoptosis rates of 19.15% ± 0.65% and 6.80% ± 0.53%, respectively. Similarly, Rg3 (S) through liver metabolism in A549 cells reported an increased early and late apoptosis rates of 42.12% ± 0.44% and 7.66% ± 0.38%, respectively (Figures 5A, B). After hepatic metabolism, Rg3 (S) promoted the early apoptosis of A549 cells and increased the apoptosis rate.

The three ginsenosides increased the apoptosis rates predominantly by promoting the early apoptosis of MCF-7 (Figure 5C). CK, Rh2 (S), and Rg3 (S) metabolized by liver significantly increased the apoptosis rate of MCF-7 cells (Figure 5D). Particularly, Rg3 (S) without the effect of liver metabolism on MCF-7 reported an early apoptosis rate of 18.15% ± 0.81%, which increased to 45.79% ± 0.54% after liver metabolism.

Figures 5E, F show the effect of ginsenosides on apoptosis of HL7702 cells. Similar to the results of MCF-7 cells, CK, Re, and Rg3 (S) promoted early apoptosis, increasing the apoptosis rate, which is dependent on liver metabolism. Among all ginsenosides, Rg3 (S) showed the most significant effect on promoting early apoptosis of HL7702 cells through liver metabolism.

### Metabolic characteristics of different ginsenosides in hepatocytes

**FIGURE 6:**
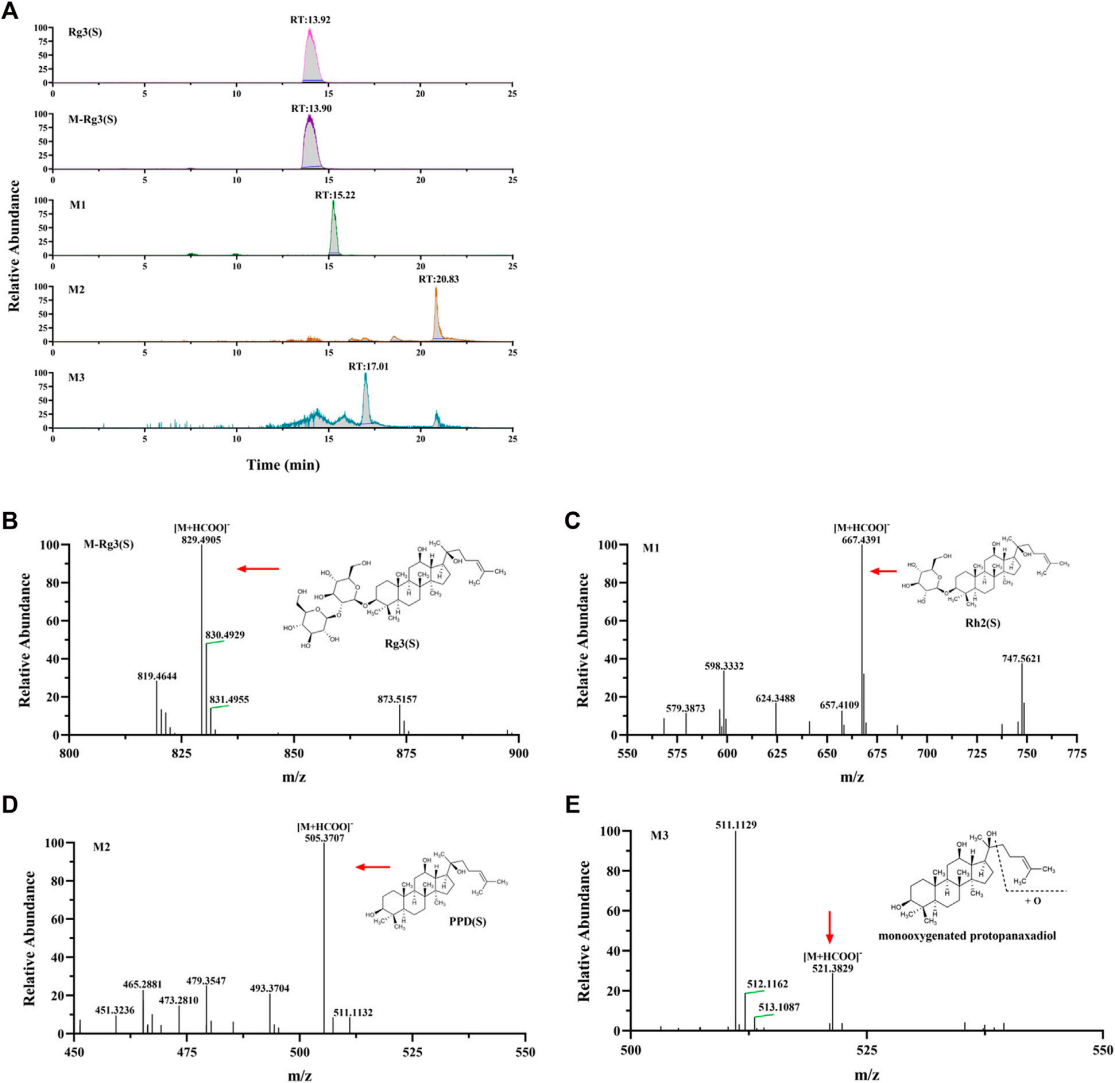
Analysis of Metabolic components of ginsenoside Rg3 (S). **(A)** Extracted ion chromatograms of prodrug and 3 metabolites in Rg3(S) sample and standard Rg3(S) sample. Characteristic peak of ginsenoside Rg3(S) **(B)**, M1 **(C)**, M2 **(D)** and M3 **(E)** in the sample.

Combined with literature, standard, and database (Qian et al., 2005a; Qian et al., 2005b; Zhang et al., 2009; Bae et al., 2013; Peng et al., 2016), the monitored metabolites were identified according to retention time and mass–charge ratio in UPLC-MS matrix (see Table 3). Rg3 (S) and three metabolites, including M1, M2, and M3, were found in the cellular metabolic fluid of Rg3 (S). The extracted ion flow diagram of the standard Rg3 (S) and metabolic samples are shown in Figure 6A, and the corresponding characteristic peaks of mass spectrometry are shown in Figures 6B–E. M1 is a Rh2 (S) ginsenoside, which was obtained by removing glucosyl at position C3 by Rg3 (S). M2 is a proto-panaxadiol-type sapogenin PPD (S), which was obtained from the conversion of Rg3 (S) to Rh2 (S) by the removal of glucosyl at position C3 by Rg3 (S). M3 is mono-oxypropanaxadiol, and its formation pathway is unknown; hence, they may be obtained by further oxidative metabolism of PPD (S) or deglycosylation of Rg3 (S) metabolites.

**TABLE 3:** Compounds identified by UPLC-MS in ginsenoside metabolites.

Number	Rt(min)	Pre cursor MZ	Pre cursor type	q value	Error(ppm)	Theoretical mass	Formula	Name	Concentration
M-Rg3(S)	13.90	829.4905	[M+HCOO]-	5.36E+06	0.0068	784.4973	C_42_H_72_O_13_	Rg3(S)	12.44 μ g/ml
M1	15.22	667.4391	[M+HCOO]-	9.00E+04	0.0054	622.4445	C_36_H_62_O_8_	Rh2(S)	81.69 ng/ml
M2	20.83	505.3707	[M+HCOO]-	7.81E+05	0.0209	460.3916	C_30_H_52_O_3_	PPD(S)	934.80 ng/ml
M3	17.01	521.3829	[M+HCOO]-	5.78E+03	0.0037	476.3866	C_30_H_52_O_4_	Monooxygenated protopannaxadiol	27.78 ng/ml
M-Rg2(S)	15.24	667.4384	[M+HCOO]-	1.47E+06	0.0061	622.4445	C_36_H_62_O_8_	Rh2(S)	1.71 μ g/ml
M4	20.85	505.3707	[M+HCOO]-	2.08E+05	0.0209	460.3916	C_30_H_52_O_3_	PPPD(S)	409.97 ng/ml
M5	14.88	683.4344	[M+HCOO]-	1.69E+04	0.0050	638.4394	C_36_H_62_O_9_	Monooxygenated RH2	16.64 ng/ml
M6	16.98	521.3826	[M+HCOO]-	7.25E+03	0.0040	476.3866	C_30_H_52_O_4_	Monooxygenated protopannaxadiol	18.41 ng/ml
M-CK	15.16	667.4384	[M+HCOO]-	2.46E+06	0.0061	622.4445	C_36_H_62_O_8_	CK	2.91 μ g/ml
M7	20.86	505.3691	[M+HCOO]-	4.75E+05	0.0225	460.3916	C_30_H_52_O_3_	PPD(S)	609.54 ng/ml

**FIGURE 7:**
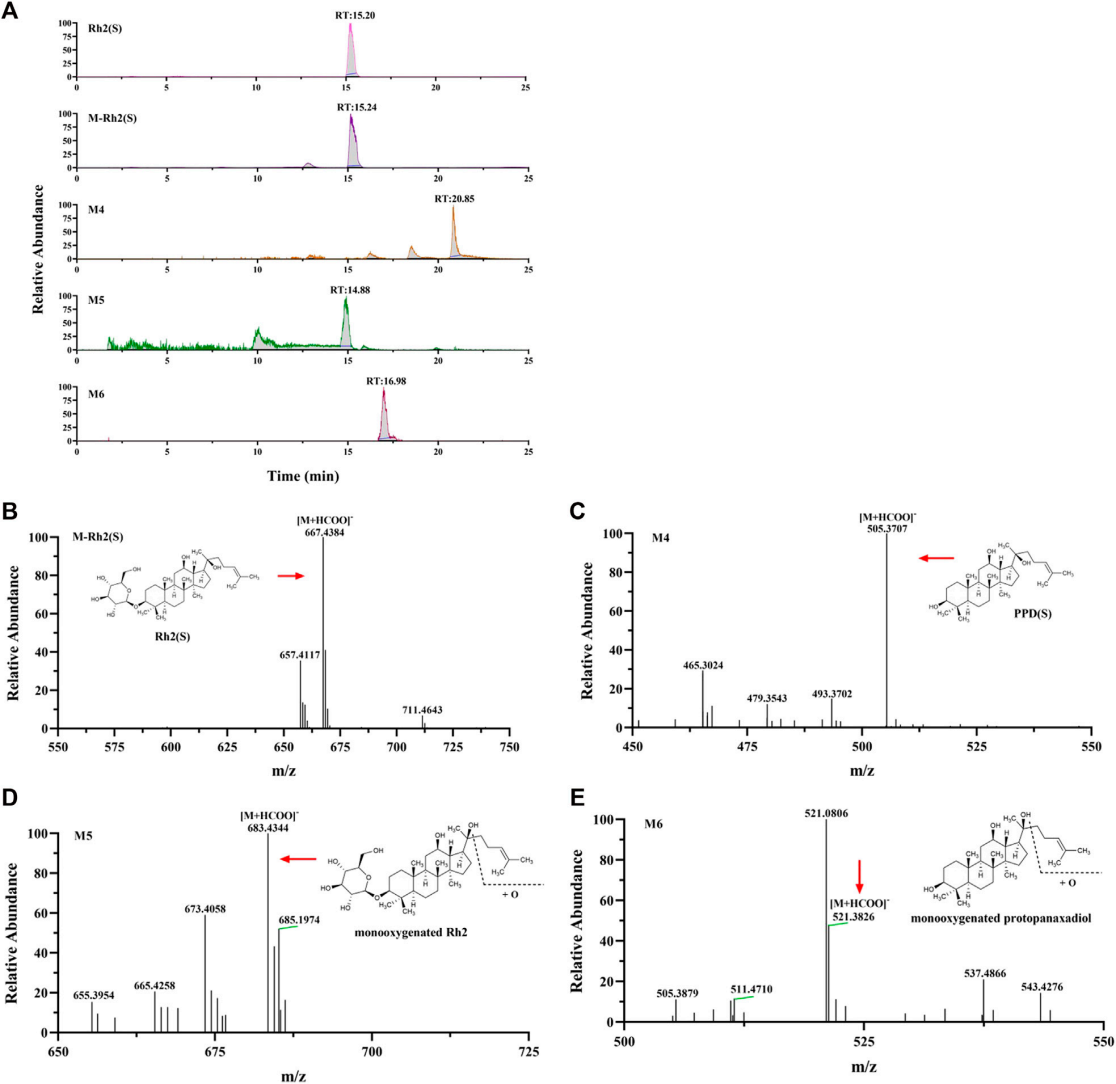
Analysis of Metabolic components of ginsenoside Rh2 (S). **(A)** Extracted ion chromatograms of prodrug and 3 metabolites in Rh2(S) sample and standard Rh2(S) sample. Characteristic peak of ginsenoside Rh2(S) **(B)**, M4 **(C)**, M5 **(D)** and M6 **(E)** in the sample.

Rh2 (S) and three metabolites, including M4, M5, and M6, were found in the cellular metabolic fluid of Rh2 (S). The extracted ion flow diagram of the standard Rh2 (S) and metabolic samples are shown in Figure 7A, and the corresponding characteristic peaks of mass spectrometry are shown in Figures 7B–E. M4 is a proto-human panaxadiol sapogenin PPD (S), which was obtained by removing glucosyl from the C3 position by Rh2 (S), and M6 is a mono-oxypropanaxadiol. M5 is a mono-oxyginsenoside Rh2, a direct oxidative metabolite of Rh2 (S).

**FIGURE 8:**
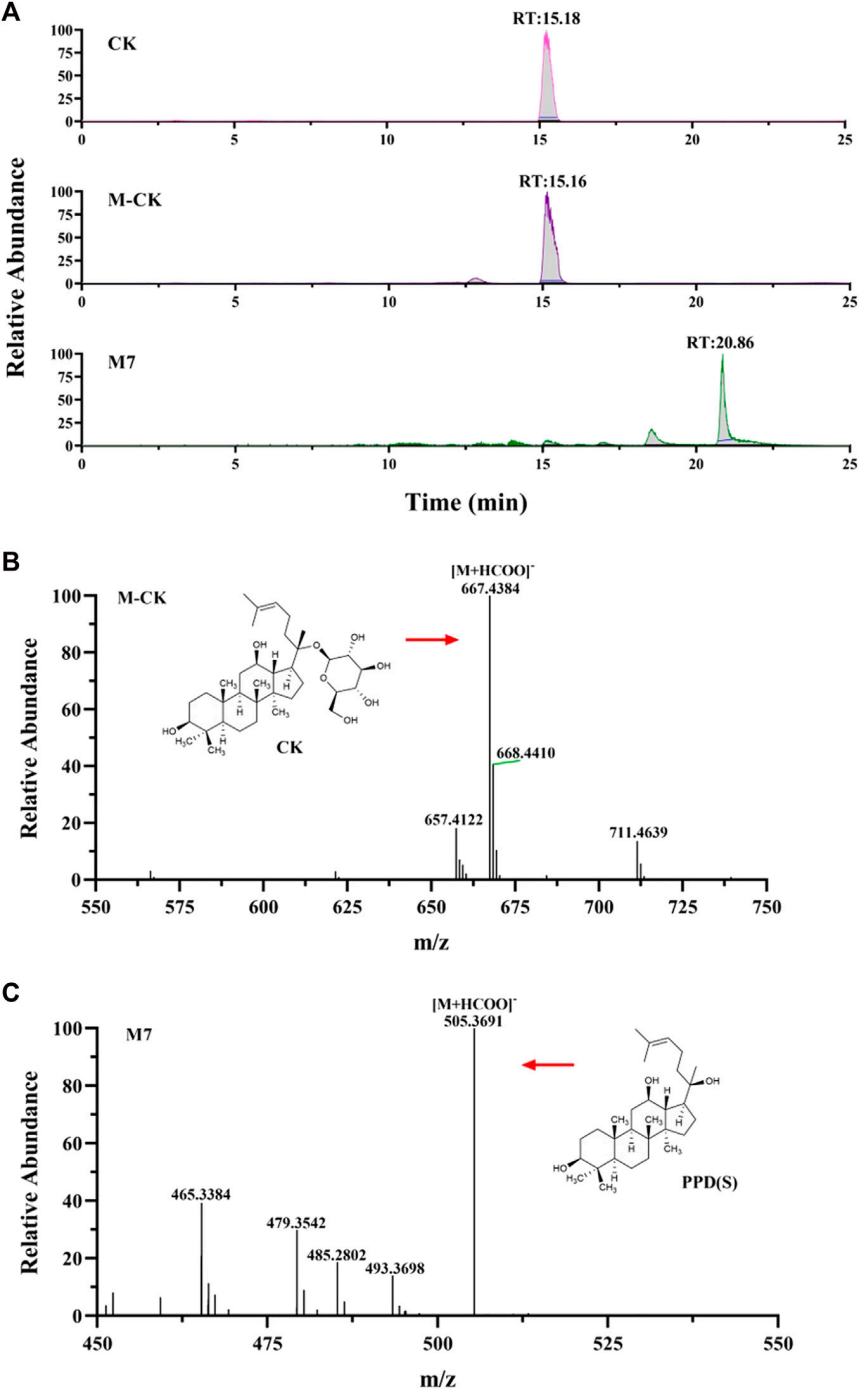
Analysis of Metabolic components of ginsenoside CK. **(A)** Extracted ion chromatograms of prodrug and metabolites M7 in CK sample and standard CK sample. Characteristic peak of ginsenoside CK **(B)** and M7 **(C)** in the sample.

CK and metabolite M7 were found in the cellular metabolic fluid of CK. The extracted ion flow diagram of standard CK and metabolite M7 is shown in Figure 8A, and the corresponding characteristic peaks of mass spectrometry are shown in Figures 8B, C. Comparing the ion flow diagrams, the retention time of M2, M4 and M7 were 20.83, 20.85 and 20.86 min, respectively. The difference in retention time among the three metabolites is < 0.1 min, indicating that M2, M4 and M7 are the same compound, namely protopanaxadiol sapogenin PPD(S). M7 was obtained from CK by removing glucosyl at C20 position.

## Discussion

Ginsenosides are primary constituents of ginseng, which are metabolized through predominantly hepatic metabolism *in vivo* (Jin L et al., 2018). *In vivo*, the metabolism dictates the composition of ingested drug, thereby affecting its pharmacological and clinical efficiency (Kv A et al., 2022). In drug development, the discovery of active metabolites may lead to a new and improved drug candidate, while metabolic inactivation of the drug may requires further optimization. Therefore, determining the metabolic profile of ginsenosides in anticancer is essential. Most studies on ginsenoside metabolism are based on rats (Lm A et al., 2022, Liu Z et al., 2021). However, the metabolic profiles of most drugs in rodents and humans are incomparable due to the difference in species. The present study is the first *in vitro* study on ginsenoside metabolism and biological activity in organs-on-chip.

In this study, an integrated microdevice mimicked the *in vivo* process of hepatic metabolism upon drug exposure. HepG2 cells representing the liver were cultured on the top layer of the chamber, due to this cell type could generate the required enzymes analogous to humans in the study of drug metabolism (Matthieu et al., 2014; Yang et al., 2014; Stampella et al., 2015; Tang et al., 2015; Zhang et al., 2015; Cui et al., 2016). In the pre-experiment, the metabolic function of HepG2 cells satisfied the experimental purpose compared with other hepatocytes. The two-layer design of organs-on-chip added viewing window performed satisfactorily with functionality and flexibility. It constructed a well-organized system with multi-cell compartments in a single device, and effortlessly facilitated the assessment of drug metabolism and anticancer activity on multiple conditions. At the same time, we using Capecitabine as a positive control, which is a common chemotherapeutic agent. The data showed a metabolism dependent drug efficacy of CAP in this system as existing *in vivo*, which demonstrated the model is validated and controllable.

Furthermore, the effect of ginsenoside exposure on the cytotoxicity of various cells on this chip was characterized. Some studies have confirmed that ginsenosides can inhibit the proliferation of human lung cancer cell lines and breast cancer cell lines. For example, the IC50 values of ginsenosides Rg3 (S), Rh2 (S), PPD and 25-OH-PPD on A549 cells are 264.6 μM, 33.9 μM, 27.2 μM, 22.5 μM, respectively, and on MCF-7 cells are 361.2 μM, 41.5 μM, 68.4 μM, 59.8 μM (Wang et al., 2007; Yu et al., 2018). However, the IC50 values of Rh2 (S) to A549 and MCF-7 are different in different studies. Qu et al. found that the IC_50_ value of Rh2 (S) on A549 cells was 12.45 μM (Qu et al., 2018). Kim et al. reported the inhibition rate of MCF-7 by 80 μmol/L Rh2 for 24 h was 59.98% (Kim, 2018). In addition, another study reported that when CK was 70 μM, the inhibition rate of MCF-7 cells treated for 24 h was 59% (Kwak et al., 2015). In this study, high concentration (100 μM) of Rg3 (S) had no significant inhibitory activity on all cells. The inhibition rate of MCF cells treated with 50 μM CK for 24 h was 20.4%, and when the concentration increased to 100 μM, the inhibition rate was more than 90%. These results are consistent with the literature. As shown in Figure 2C; Figure 3C; Figure 4C, the half-inhibitory concentration of Rh2 (S) on three kinds of target cells ranges from 50 μM to 100 μM, which is slightly higher than that reported in the literature. The biological activity of ginsenoside Re is mainly manifested in anti-diabetes, neuroregulation, anti-inflammation, protection of cardiovascular system and other aspects (Gao et al., 2022), but has no significant inhibitory effect on tumor cells, which is also reflected in the cytotoxicity results of this study.

**FIGURE 9:**
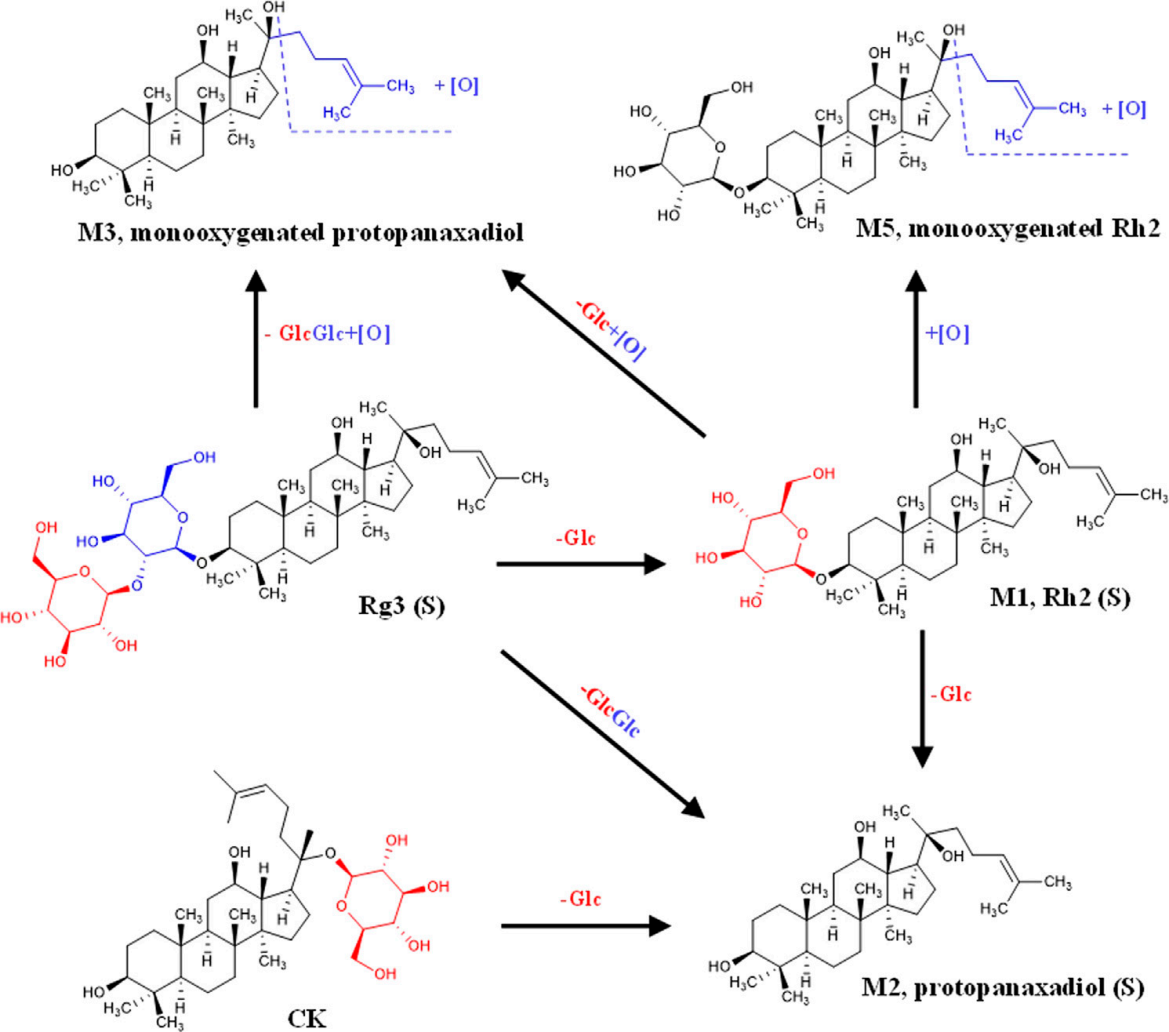
Liver metabolic pathway of ginsenosides Rg3(S), Rh2(S) and CK in liver-tumor co-culture system.

In our current work, we demonstrated that liver metabolism significantly enhanced the inhibitory effect of high concentration (100 μM) ginsenoside Rg3 (S) on A549, MCF-7 and HL7702 cells. Apoptosis experiment further confirmed that the liver metabolites of Rg3 (S) can promote early apoptosis. The metabolites were detected. As shown in Figure 9, part of Rg3 (S) was converted into Rh2 (S), PPD (S) and monooxygen PPD (S). The results of cytotoxicity of ginsenosides showed that the anti-tumor activity of 50 μM Rh2(S) and PPD(S) was significantly stronger than that of 100 μM Rg3 (S). This is also consistent with the previous literature reports that IC50 values of Rh2 (S) and PPD against A549 and MCF-7 are much smaller than Rg3 (S). The study on the structure–activity relationship between ginsenoside monomers and antitumor activity showed that ginsenosides and aglycones with a low-sugar chain had strong antitumor activity in the following order: Aglycones > monosaccharides > disaccharides > trisaccharides > tetrasaccharides (Attele et al., 1999). Therefore, after liver metabolism, Rg3 (S) removes the C3 glycosyl group and converts it into Rh2 (S) and aglycone PPD (S), which may be the reason for its enhanced anti-tumor activity. Recent studies have shown that Rg3 (S), Rh2 (S) and PPD (S) can all promote apoptosis, and at the same concentration, the promoting effect of Rh2 (S) and PPD (S) is stronger than that of Rg3 (S) (Zhang et al., 2013; Joo et al., 2015; Song et al., 2022). This may make the liver metabolites of Rg3 (S) have a stronger effect on promoting apoptosis than Rg3 (S). High concentrations of CK and Rh2 (S) had strong inhibitory effects on the three kinds of cells, regardless of whether they were metabolized through the liver or not. This may be because high concentrations of CK and Rh2 (S) inhibit the activity of hepatocytes and affect their metabolic function, resulting in most of CK and Rh2 (S) directly acting on the target cells through the porous membrane. Interestingly, we found that the liver metabolized Rh2 (S) inhibited the apoptosis of A549 cells, which may be due to the change of biological activity after part of Rh2 (S) was converted into monooxygenic Rh2 (S). Although the biological activity of ginsenoside has been studied thoroughly, the activity of ginsenoside metabolites, especially oxides, needs to be further studied. *In vivo*, ginsenosides may form new metabolites after metabolism, changing their biological activity. Consistent with the activity responses to ginsenosides, cell viability was greatly altered upon exposure to liver-on-a-chip. Distinctly, the result suggested that liver metabolism may modify the activity of the drug. These data suggest that the system could allowed us to, at least partially, reproduce hepatic drug metabolism and assess the biological activity of drugs. Thus, the system mimicked the complex physiological events associated with drug metabolism and biological activity better than conventional approaches *in vitro*.

In this study, two monooxygen metabolites of PPD-type ginsenosides (monooxygenin Rh2 and monooxypropanaxadiol sapogenin) were obtained from the liver tumor co-culture system based on microfluidic technology. Previous studies have shown that the metabolism of ginsenosides *in vivo* includes ginsenoside de-glycosylation induced by colonic bacteria and saponins oxidation mediated by cytochrome P450 enzymes (CYP enzymes) in intestine and liver (Hu et al., 2013). CYP enzymes are superfamily enzymes with hemoglobin structure, which play a key role in the metabolism of endogenous substances and drugs (Lee et al., 2022). It has been proved *in vitro* for many years that human CYP3A4 and CYP3A5 are the main enzymes responsible for the oxidation of 20 (S)–protopanaxadiol and 20 (S)–protopanaxatriol (Hao et al., 2008; Hao et al., 2010; Pintusophon et al., 2019). In addition, CYP1A2, CYP2A6, CYP2B6, CYP2C8, CYP219, CYP2D6 and CYP2E1 can also mediate the oxidation of 20 (S)-protopanaxadiol (Hu et al., 2013; Lee et al., 2022). Therefore, it is speculated that the two monooxygen metabolites obtained in this study may be related to the catalysis of these enzymes.

A high sensitivity method was required to detect and analyze the PPD metabolites, due to the characteristics of micro-quantification of organs-on-chip. UPLC-MS exhibited excellent performance for metabolite detection with high speed and high detection sensitivity (Deng et al., 2014). Rg3 (S), Rh2 (S), and CK belong to the protopanaxadiol saponin group. The metabolic pathways of the three saponins are shown in Figure 9. These ginsenosides undergo orderly deglycemic metabolism and oxidation during liver metabolism. The content of PPD (S) in the metabolites of the three ginsenosides was the highest, indicating that PPD (S) was the main metabolite of Rg3 (S), Rh2 (S), and CK through the liver (Table 3). The results of metabolite determination were consistent with the previous studies on the metabolism of Rg3 in rat liver S9 *in vitro* (Cai et al., 2003), and also consistent with the previous reports on Rg3 (Peng et al., 2016) and Rh2 (Qian et al., 2005a) *in vivo*. In addition, ginsenoside PPD and its monooxides were detected in human plasma after intravenous injection of Panax notoginseng extract containing Rg3 and Rh2 in 24 volunteers (Hu et al., 2013). The same metabolites were also obtained in this study, indicating that our liver-tumor co-culture system based on microfluidic technology can predict drug metabolism *in vivo* to a certain extent.

In summary, a liver-tumor co-culture system was constructed successfully based on the microfluidic technology. The proposed approach can be applied in reliable drug testing in an *in vivo*-like manner, indicating the potential of this device for drug screening applications. The effect of hepatic metabolism was investigated on the anticancer activity of ginsenosides, the biological activity of ginsenosides was compared, and changes in the chemical composition of the original ginsenoside diol-type saponins after hepatic metabolism were investigated, providing a rational application and scientific development of ginsenosides and their derivatives for anticancer.

## Data Availability

The original contributions presented in the study are included in the article/Supplementary Material, further inquiries can be directed to the corresponding authors.
